# Supplementary material for: Early and late onset pre-eclampsia and small for gestational age risk in subsequent pregnancies
Source: PLoS One. 2020 Mar 27;15(3):e0230483. doi: 10.1371/journal.pone.0230483 (PMC7100959; doi:10.1371/journal.pone.0230483)
Supplement: Attachment — Submitted filename: Response to Reviewers.docx [file pone.0230483.s001.docx]

**Reviewer #1**

**Q1. Alternating the care between the Dutch perinatal caregivers, general practitioners and midwives cannot be ruled out. Moreover, is it possible that the higher risk population's care will be provided by the perinatal caregivers as opposed to midwifery care, for example?**

Answer: Yes, as described in the first paragraph of the Methods section, the Perined national Dutch registry is composed of linked data from three different registries: the midwifery registry (LVR1), the obstetrics registry (LVR2), and the neonatology registry (LNR). As a result, it encompasses 96% of all deliveries that occur in the Netherlands.

In a population-based cohort study it is to be expected that care is provided through all levels of complexity and risk as represented in the health system under study. This study is no exception and reflects the particularities of obstetric care in the Dutch system. Primary care is offered by midwives and general practitioners, who refer high risk women to secondary and tertiary care, representing general and academic hospitals, respectively. Overall, we feel that our data are a good reflection of the case-mix and care system for pregnant women in The Netherlands.

**Q2. Data regarding other related factors such as BMI and other important risk factors for preeclampsia such as vascular, kidney of autoimmune disease was not provided and not controlled for.**

Answer: As mentioned in our Discussion section under the “Strengths and limitations” sub-heading:

*“Perined records do not include or generally underreport additional confounders that would further enrich these analyses such as BMI, smoking, medication use, pre-existing vascular and kidney disease, history of thrombophilia, paternal influence and family history of PE”.*

We agree that access to this information would be helpful for the analyses, but they are unfortunately not available in the population-based dataset.

**3. Pre-gestational diabetes is a potential confounder and an important risk factor for preeclampsia and was not controlled for in the multivariable logistic regression model.**

Answer: Pre-gestational diabetes was included as a potential confounder and controlled for in the multivariable logistic regression models as stated in our Methods section. We have noticed that in the same section we did not mention pre-gestational diabetes as a variable that we have controlled for in that section. Consequently, we have added this information to the relevant sentence and it now reads as follows:

*To adjust for potential confounders the following 1^st^ pregnancy clinical and demographic characteristics were included in the regressions: maternal age (years), gestational age at delivery (weeks and before/after 34 weeks of gestational age), non-Caucasian ethnicity (yes or no), low socioeconomic status (yes or no), any cause hypertension (yes or no), pre-eclampsia (yes or no), chronic hypertension (yes or no), pre-gestational diabetes (yes or no), gestational diabetes (yes or no), placental abruption (yes or no), HELLP syndrome (yes or no), assisted reproduction (yes or no), spontaneous labor (yes or no), stillbirth (yes or no), neonatal mortality (yes or no), congenital abnormalities (yes or no).*

**Q4. No description of those lost to follow up between consecutive pregnancies was provided.**

Answer: By construction, as a result of the probabilistic record linkage procedure used to match 1^st^ and 2^nd^ deliveries, there is no loss to follow up for the women included in the linked dataset. If the calculated posterior probability of a proper match between 1^st^ and 2^nd^ deliveries was higher than 80% it was considered high enough to assume it belonged to the same mother. Full description of the methods used in this procedure are referenced in the manuscript and found here:

Schaaf JM, Hof MHP, Mol BWJ, Abu-Hanna A, Ravelli ACJ. Recurrence risk of preterm birth in subsequent singleton pregnancy after preterm twin delivery. Am J Obstet Gynecol. 2012 Oct;207(4):279.e1-7.

**Reviewer #2**

**Q1. In figure 1, the increased risk of SGA (both p10 in the first pregnancy? Or is the increased risk of SGA in the subsequent pregnancy merely the result of recurrence of SGA in these groups? This should be explained more clearly or added in the analysis.
Hereby, it should also be taken into account that birthweight charts are influenced by the association between placental pathology and preterm delivery. Consequently the amount of SGA infants tends to be underestimated in the preterm period.**

Answer: Results presented in figure 1 correspond to the multivariable logistic regression models that evaluate 2^nd^ delivery risk of SGA infants based on data of the 1^st^ pregnancy. In particular, we were interested in assessing the effects of pre-eclampsia, hypertension and gestational age at delivery on this risk. To more accurately find the effect-sizes of each particular factor, we included in the model variables such as delivery of an SGA infant in the 1^st^ pregnancy, a well-established risk factor for subsequent SGA delivery.

The advantage of using a multivariable model in the evaluation of multifactorial issues such as the delivery of an SGA infant is that we can control for the influence of factors we have data available for. In this sense, the increased risk of SGA in the 2^nd^ pregnancy associated with SGA delivery in the 1^st^ pregnancy found in figure 1 represents the risk associated with this factor controlled for pre-eclampsia, hypertension, delivery before or after 34 weeks and all the other factors listed in our Methods section such as maternal age, diabetes (chronic and gestational) and congenital abnormalities. This follows for all other variables in the model.

The influence of pathology and preterm delivery on birthweight is the *raison d'être* for the construction of birthweight charts and definitions such as SGA. With this in mind, the inclusion of variables such as congenital abnormalities and placental abruption as variables in our models no doubt adds strength to the results.

**Q2. Also I suggest that for clinical use, some findings should/could be highlighted more clearly. For both patients and clinicians absolute risks are more useful than relative risks or odds ratios.**

Answer: We agree to an extent and that is why we have provided absolute risks in Figures 1 and 2. We believe though that these should be interpreted in the wider context of a multifactorial problem and that requires the inclusion of estimates such as odds ratios adjusted in a multivariable model.

**Q3.** **"Women with 1st pregnancy early onset pre-eclampsia have increased risk of SGA <5th percentile
45 in the 2nd pregnancy." is not complete/correct. This should be something like: "Women with 1st pregnancy early onset hypertension, pre-eclampsia or delivery <34 weeks have increased risk of SGA (<5th and 5th-10th percentile) in the 2nd pregnancy."**

Answer: Although we appreciate the suggestion, we prefer to limit our abstract conclusion to refer specifically to the findings that pertain to our original objectives. These were defined in the abstract as:

*To investigate whether delivery of a small for gestational age (SGA) infant in the 1st pregnancy increases the risk of early and late onset pre-eclampsia in the 2nd pregnancy. Conversely, we investigated whether pre-eclampsia in the 1st pregnancy impacts SGA risk in the 2nd pregnancy.*

**Q4. conclusion: "SGA in the 1st pregnancy increases pre-eclampsia risk in the 2nd pregnancy even in the absence of hypertensive disorders in the 1st pregnancy" should in my opinion be adjusted: "SGA in the 1st pregnancy increases pre-eclampsia risk in the 2nd pregnancy even in the absence of hypertensive disorders in the 1st pregnancy the is an increased risk of pre-eclampsia in the 2nd pregnancy if the first born had a birthweight below the 5th percentile... however, absolute risks are very low (<0.1% for early onset pre-eclampsia, and <0.7% for late onset pre-eclampsia)"**

Answer: We appreciate the suggestion and have adjusted the abstract conclusion as follows:

*SGA in the 1st pregnancy increases pre-eclampsia risk in the 2nd pregnancy even in the absence of hypertensive disorders in the 1st pregnancy, although absolute risks remain low.*

**Q5.Introduction
Line 51: "The severity of adverse outcomes has strong association..." ==> consider changing to: "The severity of adverse outcomes has a strong association..."**

Answer: We appreciate the suggestion and have adjusted the introduction accordingly.
